# The influence of larval competition on Brazilian *Wolbachia*-infected *Aedes aegypti* mosquitoes

**DOI:** 10.1186/s13071-016-1559-5

**Published:** 2016-05-16

**Authors:** Heverton Leandro Carneiro Dutra, Vanessa Lopes da Silva, Mariana da Rocha Fernandes, Carlos Logullo, Rafael Maciel-de-Freitas, Luciano Andrade Moreira

**Affiliations:** Mosquitos Vetores: Endossimbiontes e Interação Patógeno-Vetor, Centro de Pesquisas René Rachou - Fiocruz, Belo Horizonte, MG Brazil; Laboratório de Transmissores de Hematozoários, Instituto Oswaldo Cruz, Fiocruz, Rio de Janeiro, RJ Brazil; Laboratório de Sanidade Animal, Laboratório de Química e Função de Proteínas e Peptídeos e Unidade de Experimentação Animal - RJ, UENF, Campos dos Goytacazes, Rio de Janeiro Brazil

**Keywords:** *Wolbachia*, *Aedes aegypti*, Development time, Larval competition, Morphometrics, Glycogen

## Abstract

**Background:**

With field releases starting in Brazil, particular interest must be given to understanding how the endosymbiotic bacterium *Wolbachia pipientis* affects *Aedes aegypti* mosquitoes with a Brazilian genetic background. Currently, there is limited information on how the bacterium affects phenotypic traits such as larval development rate, metabolic reserves and morphometric parameters in *Ae. aegypti*. Here, we analyze for the first time, the effect of *Wolbachia* on these key phenotypes and consider how this might impact the potential of the bacterium as a disease control agent in Brazil.

**Methods:**

We examined the influence of the *w*Mel strain of *Wolbachia* in laboratory *Ae. aegypti* with a Brazilian genetic background, reared under different larval densities. Pupae formation was counted daily to assess differences in development rates. Levels of metabolic reserves and morphometric parameters were assessed in adults resulting from each larval condition.

**Results:**

*w*Mel infection led to more rapid larval development at higher densities for both males and females, with no effect under less crowded conditions in females. Infection also led to reduced body size at both high and low density, but not at intermediate density, although the scale of this difference was maintained regardless of larval density, in comparison to uninfected individuals. Wing shape also varied significantly between infected and uninfected mosquitoes due to larval density. Glycogen levels in uninfected mosquitoes decreased under higher larval density, but were consistently high with *Wolbachia* infection, regardless of larval density.

**Conclusions:**

We demonstrate that the *w*Mel *Wolbachia* strain can positively influence some important host fitness traits, and that this interaction is directly linked to the conditions in which the host is reared. Combined with previously published data, these results suggest that this *Wolbachia* strain could be successfully used as part of the Eliminate Dengue Program in Brazil.

**Electronic supplementary material:**

The online version of this article (doi:10.1186/s13071-016-1559-5) contains supplementary material, which is available to authorized users.

## Background

Mosquitoes of the family Culicidae are considered the most important group of insects involved in disease transmission in humans. The mosquito *Aedes aegypti* is primarily responsible for the transmission of dengue, the most prevalent mosquito-borne viral disease [1], which has seen a 30-fold increase in incidence over the last half century, dramatically increasing the burden on human health [2]. The transmission cycle of dengue and other vector-borne diseases depends on the existence of a sophisticated tripartite relationship between the pathogen and invertebrate and vertebrate hosts. Vector competence in mosquitoes is dependent on a wide variety of extrinsic and intrinsic factors [3, 4].

Dengue transmission is therefore strongly linked to host fitness and physiology. As such, environmental conditions during larval development can affect many important life history traits during adulthood, including fecundity, fertility, immune response and host seeking [5–7]. These environmental conditions include the availability of nutritious particles dissolved in the water of the breeding sites that larvae use as food, the influence of temperature, and crowding condition itself [8]. Competition for these resources between larvae can detrimentally affect development time [9] and the levels of key adult energy reserves such as lipids or glycogen [10], the primary carbohydrate reserves in insects [11]. Glycogen is the principal energetic component utilized by mosquitoes to fuel flight [10], particularly over short distances, as undertaken during host-seeking by female mosquitoes [12]. The conditions experienced during larval development directly influence adult mosquito size and fitness, with smaller mosquitoes experiencing impairments in blood-feeding [13], flight [14] and mating performance [15]. Smaller mosquitoes also display a high susceptibility to infection, increased dissemination rate of dengue virus (DENV) infection [16] and have altered host gene expression [17], all of which affect mosquito vector competence [18]. As such, larval competition, commonly present in nature [19–21], plays a major role in shaping the vectorial capacity of a mosquito species.

*Wolbachia pipientis* (hereafter *Wolbachia*) is amongst the most widespread endosymbiotic bacteria known [22]. This species is currently being evaluated as a novel biocontrol agent to reduce dengue transmission, by exploiting some of the many phenotypic changes the bacteria make to their host’s biology [23]. *Wolbachia*’s effects can range from fitness costs [24–27] to fitness benefits [28, 29]. *Wolbachia* is not naturally present in *Ae. aegypti* [30], but several strains have been artificially introduced into this mosquito via the process of transinfection [31, 32]. Critically and encouragingly from the perspective of disease control, *Wolbachia* limits infection by key pathogens in the mosquito host, including dengue virus [32–34]. Consequently, it represents a promising biocontrol agent that is currently being used in population replacement strategies around the world [32, 35–37].

There are many physiological changes associated with *Wolbachia* infection in adult *Ae. aegypti*, some of which have an associated fitness cost [38, 39]. However, there is relatively little information on the role played by *Wolbachia* in the development of immature mosquitoes and how this might contribute to adult fitness. Any potential fitness effects from altered development in *Wolbachia*-infected mosquitoes must be considered when using the bacterium as a biocontrol agent, as it could affect their ability to compete against wild type mosquitoes in the field, and thus hamper bacterial spread [40]. In Australian *Ae. aegypti* mosquitoes, the *w*MelPop *Wolbachia* strain causes infected larvae to develop faster than uninfected individuals under low nutritional conditions and high larval density, whereas the opposite effect occurred when food availability was high and larval density low [39]. Both the *w*Mel and *w*MelPop strains caused a delay in larval development time compared to uninfected larvae in mixed cohorts of infected and uninfected individuals [25].

*Wolbachia* infection can affect levels of key nutritional reserves, as seen with the native *w*Flu strain in the mosquito *Aedes fluviatilis*, where levels of glycogen were higher in infected mosquitoes [41]. *Wolbachia* infection can also lead to altered adult body size, with *w*MelPop causing a decrease and *w*Mel an increase in comparison to uninfected mosquitoes [25]; however, this was specifically linked to the conditions in which the experiments were performed, given that a further set of experiments saw no effect of *Wolbachia* in the size of mosquito larvae reared under extremely nutritionally deprived conditions [42].

Releases of *w*Mel-infected *Ae. aegypti* have recently commenced in Brazil as part of the Eliminate Dengue Program. These mosquitoes possess a Brazilian genetic background, different to that of mosquitoes used in previous releases in Australia [43]. It is possible that different host genetic backgrounds could influence the fitness effects caused by *Wolbachia*, hindering the spread of this bacterium in the field, or induce fitness benefits that could actually enhance the chance of successful population invasion [29, 44]. So far, the effect of *w*Mel on fecundity, maternal transmission and cytoplasmic incompatibility (CI) in Brazilian *Ae. aegypti* have been characterized, where the *w*Mel strain, similarly to what was observed in the Australian genetic background, causes strong CI, a high rate of maternal transmission and has no evident detrimental effect on host fecundity or fertility [37]. However, the effects of larval competition on the fitness of *Wolbachia*-infected mosquitoes are unknown. In this study, we explored how changes in larval density conditions affected both larval and adult stages of Brazilian *Wolbachia*-infected *Ae. aegypti*. We measured the effect of *w*Mel infection on larval development time at different densities, quantified levels of the key energetic reserve metabolite glycogen in individual adult females, and then performed geometric morphometric analyses to assess the impact of *Wolbachia* infection and larval density on adult size and wing shape. Our results offer new insight into the role that host genetic background plays in *Wolbachia*-infected mosquitoes.

## Methods

### Mosquito colony maintenance

All *Ae. aegypti* mosquitoes used in our experiments were maintained in a climate controlled insectary under previously described conditions [37]. All experiments involved two previously described mosquito lines; the first (*w*Mel_Br) was generated by introducing the *w*Mel *Wolbachia* strain into a Brazilian genetic background. The second line (*w*Mel_BrTET) was cured of its *w*Mel infection by tetracycline treatment [37].

### Larval rearing and dietary conditions

In order to analyze the effect of crowding on phenotypic traits of *w*Mel-infected *Ae. aegypti*, we reared larvae under three different larval densities: either 10 (low density condition), 50 (intermediate density condition) or 250 (high density condition) larvae per tray. Eggs from both *w*Mel_Br and *w*Mel_BrTET lineages were hatched synchronously in separate trays containing filtered, dechlorinated water for 1 h without any source of food. After eclosion, first instar larvae were separated into small, black flowerpots (12 × 8.8 × 10 cm) containing 150 ml of filtered, dechlorinated water, according to their respective larval density conditions. In order to obtain around 60–100 adult mosquitoes per treatment per lineage for phenotypic traits, we raised larvae in 40, 10 or 5 trays, for the low, intermediate and the most crowded conditions, respectively. For larval development time, we recorded pupae formation in all trays in order to analyze similar number among treatments, i.e. around 350 individuals per biological replicate. For adult phenotypic traits, we randomly selected a specific number of individuals within a pool made out of each treatment (range of individuals tested is described in each specific experimental section below). Relevant to state, is the fact that we did our analysis on the larval level rather than the tray level, thus ignoring the issue of variation between trays, i.e. we compared a similar sample size.

Larvae were fed Tetramin Tropical Fish Food flakes (Tetra) as a food source. The highest level of food provided was 0.25 mg of food per larvae, each day, as previously described [39]. All trays received 2.5 mg of food per day, with the low density condition functioning as the control treatment, with these larvae receiving the highest amount of food. The other two conditions represented increasing stress due to larval competition for food, with the intermediate and high density treatments receiving 5 and 25 times less food than the low density condition, respectively. We measured larval development for each density condition by recording the time from egg hatch to pupation. Trays were examined every 24 h, where pupae were removed and sexed by visual analysis of their terminalia using a stereomicroscope.

### Morphometrics

To determine if *Wolbachia* infection and larval density affected mosquito morphometric traits, we randomly collected a total of 455 adult female mosquitoes within 24 h of emergence. These samples were collected across all treatments and from two independent biological replicates (60–100 mosquitoes measured per treatment) although not all individuals were analyzed. These mosquitoes were stored at room temperature in 70 % ethanol for later analysis, where we randomly selected approximately the same number of female mosquitoes as stated above, separately, from each biological replicate. The right wings from all mosquitoes were detached and the scales were removed manually with a paintbrush, as scales can interfere with landmark data acquisition during wing measurement [45]. Wings were mounted on microscope slides and photographed at 30.2× magnification using a Carl Zeiss AxioCam MRc camera coupled to Carl Zeiss Stemi SV 6 stereomicroscope (Zeiss, Oberkochen, Germany) using AxioVision version 4.8.1.0 image capturing software (Zeiss).

**Fig. 1:**
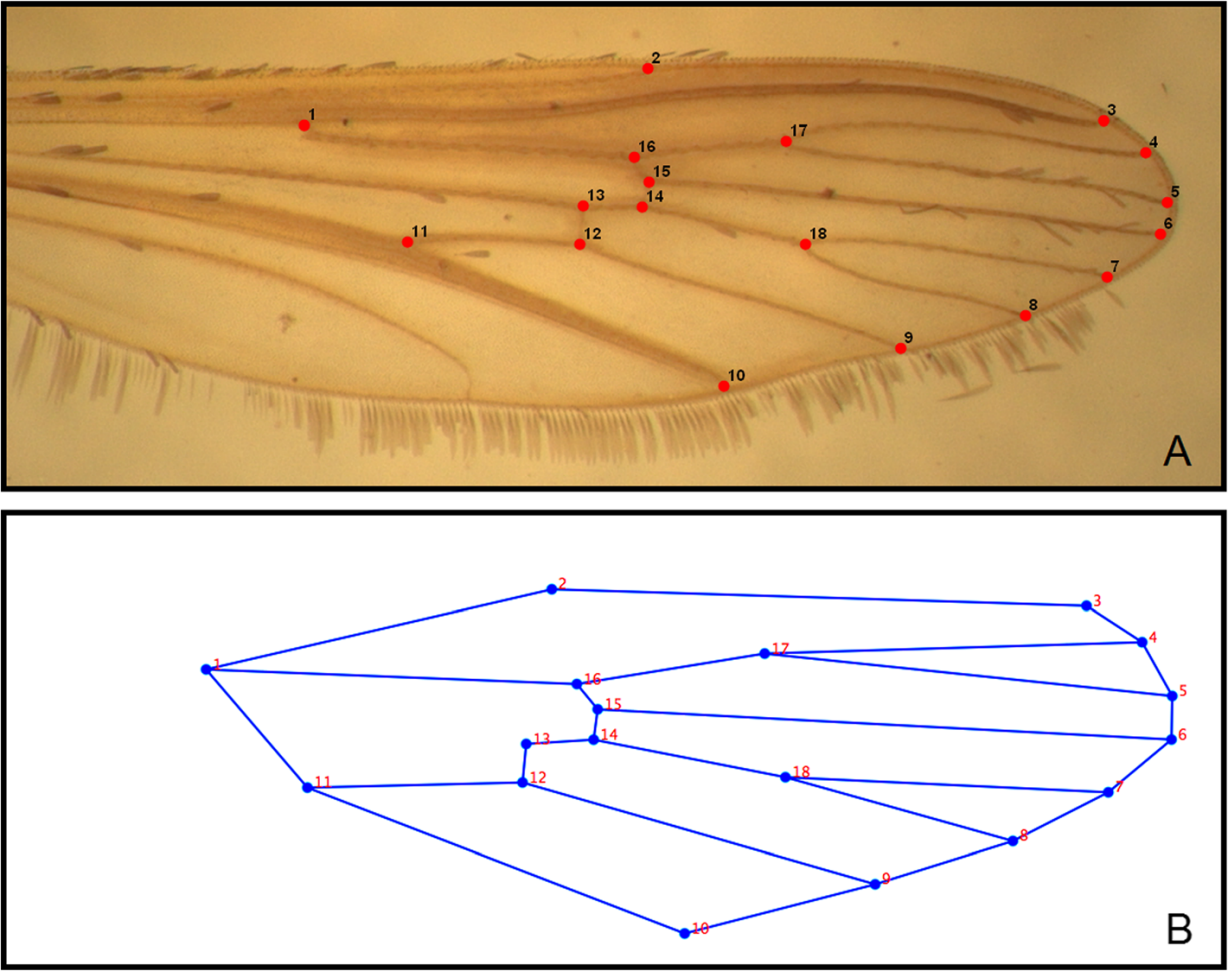
*Aedes aegypti* landmarks. **a** The right wing of an adult Brazilian female *Aedes aegypti* mosquito with its scales manually removed, showing the position of the 18 landmarks (*red dots*). **b** Scheme of the imaginary links between the 18 landmarks used to depict the consensus wing size and shape

Landmark acquisition was performed as previously described [46]. Briefly, 18 landmarks were located on each wing and digitalized using TPsDig2 version 2.17 (Fig. 1). These digitalized wing images were then plotted onto a Cartesian plane in order to produce measurements of centroid size (a proxy of wing size, see [47]) and geometric descriptors of wing shape. In order to assess body size, we took isometric measurements of centroid size, defined as the square root of the sum of squares of the Euclidean distances between landmarks to the centroid, on the right wing of each female mosquito [48]. Variations in wing shape, called partial warps, were identified by the generalized Procrustes superimposition analysis, in order to eliminate differences due to position, orientation and size [49]. The resulting superimposed dataset with covariance matrices related to principal component parameters were used to maximize separation between groups in a subsequent canonical variate analysis, which determined whether pre-defined groups can be statistically distinguished based on multivariate data.

In order to account for the potential effect of allometry (the influence of size in shape) in our analyses, we performed a multivariate regression of Procrustes coordinates against centroid size in all conditions analyzed. From the shape variables generated, wing shape differences between individuals were analyzed based on the Mahalanobis distances (MD), a method for measuring how similar a specific set of conditions is to an ideal set of conditions (defined as the square root of the distances squared between the superimposed individual to the mean shape that are standardized by the covariance matrix of the distance variables), coupled to a permutation test with 10,000 randomizations [50], where smaller MD numbers between two groups means that they are more closely related to each other.

User measurement error in data acquisition was taken into account through repeatability test procedures, described by the Pearson correlation coefficient between two measurements. This was performed by double landmarking 30 randomly chosen samples from each group using statistical tests previously described [51]. All repeatability values were above 0.99 indicating that data acquisition was accurate.

### Glycogen quantification

To determine if *Wolbachia* infection and larval density affected adult reserves, the total glycogen content of mosquitoes was quantified as previously described [41]. Briefly, a total of 300 female *Ae. aegypti* adult mosquitoes were collected from the different larval rearing conditions (*c*.40–60 mosquitoes per treatment; these mosquitoes belonged to a different cohort to that used for previous analyses of morphometric traits and development time), within 24 h of emergence and without the opportunity to feed, thus maintaining the nutritional reserves that were carried over to adulthood after pupation. These mosquitoes were homogenized individually in 200 mM sodium acetate (pH 4.8). The supernatant was removed and incubated with 1 U/ml of α-amyloglucosidase (Sigma, St Louis, MO, USA) at 40 °C for 4 h, in triplicate for each sample. This reaction produced glucose as a final product, and this was used in a colorimetric glucose quantification assay using Glucox 500 (Doles, Goiânia, Brazil). Samples were incubated for 30 min at 37 °C and then quantified using a spectrophotometer at 510 nm (Shimadzu UV-1240). As a control for our reactions, we used free glucose samples obtained from reactions without α-amyloglucosidase where the resulting values were subtracted from the experimental values in order to obtain the final amount of glycogen (i.e. reaction with enzyme - reaction without enzyme = actual value of glycogen). The glycogen content was determined using a standard curve run in parallel. Total protein levels in each mosquito were quantified using the Bradford assay. Bovine serum albumin was used to make standard curves with known concentrations of protein, which we ran in parallel with the experimental samples [52]. The glycogen content in each mosquito was normalized to total protein content, with the final glycogen data presented as a ratio of total glycogen to total protein.

### Statistics and data analysis

All assays were repeated twice on biologically independent groups of mosquitoes. The development time data were first combined for all trays in each treatment and log-probit transformed to obtain the rate of pupae formation. These data were analyzed by regression with a 95 % confidence interval in order to compare the development dynamics between groups, where dynamics refers to difference in the rate of pupation over time (peak pupae formation time). Independently, we analyzed the median development time from both groups as well as through pairwise comparisons (described below). The effects of crowding conditions and *Wolbachia* infection on development time, centroid size and glycogen levels were considered using generalized linear models of regression (SPSS V17, IBM). Pairwise effects were compared using Mann-Whitney U tests followed by multiple test correction using a false discovery rate of 0.05. For non-parametric data, Kruskal-Wallis one-way analysis of variance, followed by pairwise comparison using Dunn’s tests. Plots were made using GraphPad Prism version 6.0 g for Macintosh (GraphPad Software, San Diego, CA, USA). The morphometric statistical analyses of wing centroid size and shape (Generalized Procrustes and Canonical Variate Analysis) were performed using TpsUtil 1.60 and TpsRelw 1.53 (James Rohlf) and MorphoJ 1.06d (Flywings).

## Results

### Larval development

The effects of *Wolbachia* infection and larval density on larval development time were compared independently for males and females using generalized linear models of regression. For males and females, *Wolbachia* infection, crowding stress and the *Wolbachia* × crowding interaction had a significant influence on development time (Table 1). Interestingly, the effect of *Wolbachia* on development time changed depending on the larval density.

**Table 1:** Analysis of the effects of *Wolbachia* infection and larval density on pupal development time status using generalized linear models

Source	*W*	*df*	*P*-value	Source	*W*	*df*	*P*-value
Female development data				Male development data			
Intercept	175367.736	1	< 0.0001	Intercept	164600.498	1	< 0.0001
*Wolbachia*	37.483	1	< 0.0001	*Wolbachia*	55.389	1	< 0.0001
Crowding	12578.396	2	< 0.0001	Crowding	10864.978	2	< 0.0001
*Wolbachia* × Crowding	6.913	2	0.032	*Wolbachia* × Crowding	11.236	2	0.004

*W* Wald statistic, *df* degrees of freedom, *Wolbachia* stands for *Wolbachia* infection, crowding stands for larval density condition

**Fig. 2:**
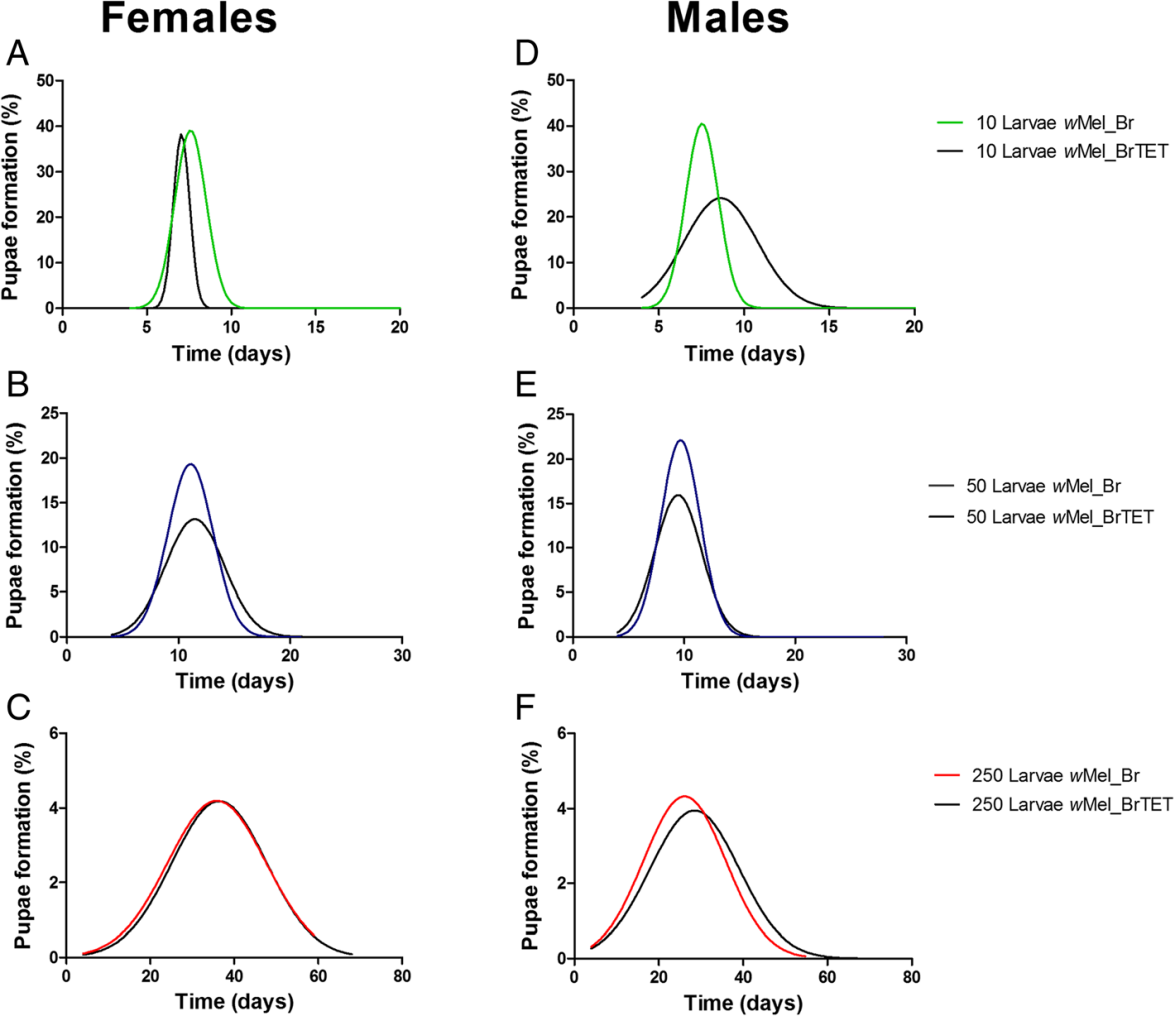
*w*Mel infection differentially affects the dynamics of pupation in male and female Brazilian *Ae. aegypti* reared under different larval densities. For females, low **a** and high larval densities **c** did not affect the pupation dynamics between *w*Mel-infected and uninfected individuals (*F*_(0.05)_ = 3.072, *P* = 0.0119) and (*F*_(0.05)_ = 0.1425, *P* = 0.9344), respectively; **b**, with differences only occurring at the intermediate condition (*F*_(0.05)_ = 3.072, *P* = 0.0119). For males, **d**, low; **e**, intermediate conditions showed differences between groups (*F*_(0.05)_ = 5.899, *P* = 0.0008) and (*F*_(0.05)_ = 2.778, *P* = 0.0457), respectively, with peaks occurring earlier, while there was no difference at **f** high density (*F*_(0.05)_ = 2.072, *P* = 0.1054). Black lines represent uninfected mosquitoes. Colored lines represent *Wolbachia*-infected mosquitoes, with a different color for each density condition. Data were pooled from two independent biological replicates

In females, for the low density condition, there was no influence of infection on development dynamics (*F*_(0.05)_ = 0.1665, *P* = 0.9189) (Fig. 2a), also, both infected and uninfected cohorts had a median pupation time of 7 days (Additional file 1: Figure S1a). In the intermediate larval condition, uninfected and infected females had statistically different development dynamics (*F*_(0.05)_ = 3.072, *P* = 0.0119) (Fig. 2b), with a median time-to-pupation of 12 days for uninfected individuals and 11 days for infected individuals (Additional file 1: Figure S1a). At high larval density, there was also no statistical difference in dynamics of development time due to infection (*F*_(0.05)_ = 0.1425, *P* = 0.9344) (Fig. 2c). There was a median pupation time of 30 and 33 days, respectively, for infected and uninfected females (Additional file 1: Figure S1a).

In males, the development dynamics were different for infected and uninfected individuals in the low density condition (*F*_(0.05)_ = 5.899, *P* = 0.0008) (Fig. 2d), however, in general, both took a median of 7 days to develop (Additional file 1: Figure S1b). Developmental dynamics also differed for the intermediate density (*F*_(0.05)_ = 2.778, *P* = 0.0457) (Fig. 2e), where there was a median pupation time of 9 and 9.5 days for infected and uninfected individuals, respectively (Additional file 1: Figure S1b). For the high-density condition, there was no difference in development dynamics (*F*_(0.05)_ = 2.072, *P* = 0.1054), but the median pupation time was 23 days for infected and 26 days for uninfected individuals (Additional file 1: Figure S1b).

### Morphometric traits

#### Size

**Fig. 3:**
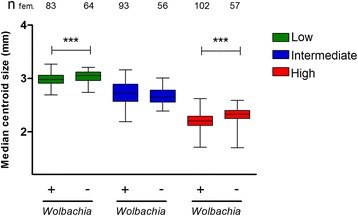
*w*Mel infection influences the median wing size of adult Brazilian *Ae. aegypti* females. Box-and-whisker plots of median wing centroid size (mm) of *w*Mel-infected (+) and uninfected (−) female mosquitoes under different crowding conditions. Green boxes represent the lower density, blue and red boxes the intermediate and higher densities, respectively. *w*Mel infection led to reduced wing size in females at the low (Mann-Whitney U test, *U* = 1811, *df* = 1, *P* = 0.0009) and high densities (Mann-Whitney U test, *U* = 1668, *df* = 1, *P* < 0.0001); however, there was no difference from uninfected females at intermediate larval density (Mann-Whitney U test, *U* = 2198, *df* = 1, *P* = 0.1119). Data were pooled from two independent biological replicates. The total number of females (n_fem._) is indicated above treatment

To obtain an estimate of the effects of *Wolbachia* infection and larval density on adult body size (Fig. 3), wing centroid size data for female mosquitoes from each treatment were compared statistically using generalized linear models of regression, where all factors included in the model were significant determinants of centroid size (Table 2). In general, wing size was decreased under high density conditions in comparison to low density conditions, with wings from uninfected mosquitoes at the higher density being 23.7 % smaller on average and 26.0 % smaller for *w*Mel-infected mosquitoes, in comparison to the lower density. Mosquitoes reared at lower densities had the largest wing size of all groups (Kruskal-Wallis *H* = 366.8, *df* = 5, *P* < 0.0001). Under low-density conditions *Wolbachia*-uninfected individuals (median = 3.05 mm; range = 2.74–3.21 mm) had wings that were 2.46 % greater than their infected counterparts (median = 2.98 mm; range = 2.69–3.27 mm), (Mann-Whitney U test, *U* = 1811, *df* = 1, *P =* 0.0009). In the intermediate condition, there was no significant difference in wing size due to infection (Mann-Whitney U test, *U* = 2198, *df* = 1, *P =* 0.1119), although the median centroid size of *Wolbachia*-infected females was 2.58 % larger than that of uninfected females. At the highest larval density, median infected mosquito wing size was 5.37 % shorter (median = 2.20 mm; range = 1.71–2.62 mm) than in uninfected females (median = 2.33 mm; range = 1.70–2.59 mm), (Mann-Whitney U test, *U* = 1668, *df* = 1, *P <* 0.0001).

**Table 2:** Analysis of the effect of *Wolbachia* infection and larval density on centroid size using generalized linear models

Source	*W*	*df*	*P*-value
Centroid size data			
Intercept	130673.435	1	< 0.0001
*Wolbachia*	5.125	1	0.024
Crowding	1860.658	2	< 0.0001
*Wolbachia* × Crowding	16.038	2	< 0.0001

*W* Wald statistic, *df* degrees of freedom, *Wolbachia* stands for *Wolbachia* infection, crowding stands for larval density condition

#### Shape

**Fig. 4:**
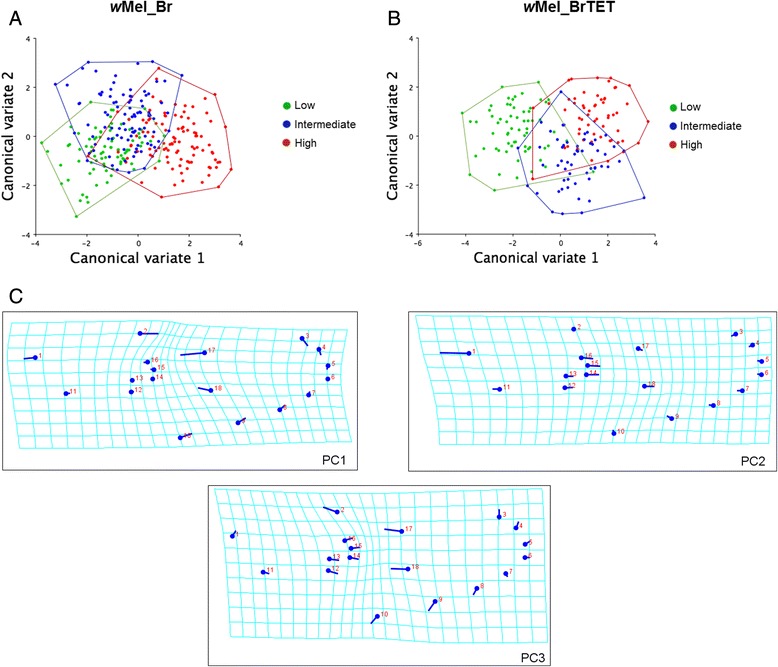
Rearing under different larval densities produced distinct wing shapes in adult Brazilian *Ae. aegypti* females. Scatterplot comparisons of wing shape for (**a**) *w*Mel_Br and (**b**) *w*Mel_BrTET female mosquitoes reared at either low (*green dots*), intermediate (*blue dots*) or high (*red dots*) larval densities, based on analysis of the main canonical variates predicting wing shape pattern. These data are depicted without allometry. Each circle represents a single adult female. Shape variations are depicted with the aid of thin-plate spline deformation grids (**c**) for PC1, PC2 and PC3, since 62.3 % of the observed variation was concentrated in these three principal components. Shape variation was scaled down 10 times in this analysis since it over-exaggerates the observed variation. Data were pooled from two independent biological replicates

The shape of morphological traits (i.e. body parts) is directly influenced by the overall body size of an individual, an effect called allometry [53]. In order to analyze the influence of size on the wing shape of female mosquitoes, we performed a separate analysis on the influence of allometry for larval density and infection. This proved to be statistically significant for both conditions and then was removed from the results. We examined wing shape separately for *w*Mel_Br and *w*Mel_BrTET mosquitoes, as there were significant differences in wing shape due to infection. For infected individuals, canonical variate analysis revealed differences in wing shape between all larval densities (Table 3), although there was a certain degree of superimposition between groups within the universe of possible organismal morphologies (called morphospace) even with the removal of the significant effect of allometry through linear regression (6.06 %; *P* < 0.001) (Fig. 4a). For uninfected individuals (Fig. 4b), as observed for infected individuals, there was a certain degree of superimposition between groups (not as high as for infected individuals); however, it was possible to distinguish between larval densities (Table 3), with allometry being significant in this group as well (8.1 %; *P* < 0.001).

**Table 3:** Mahalanobis distances (MD) for different crowding conditions without allometry

			Mahalanobis distance					
	*w*Mel_Br		*P*-value	*n*	*w*Mel_BrTET		*P*-value	*n*
Larval density	Low	Intermediate			Low	Intermediate		
Low	–	–	<0.0001	83	–	–	< 0.0001	64
Intermediate	1.53	–	<0.0001	93	2.80	–	< 0.0001	56
High	2.71	2.14	<0.0001	102	3.34	1.96	< 0.0001	57

*n* Total number of mosquito wings analyzed for each density condition

The MD between different larval densities for *w*Mel_Br and *w*Mel_BrTET is depicted in Table 3. As expected, the MD showed that there was a pattern of proximity between larval densities as the crowding condition increased for both infected and uninfected groups. Infected individuals from the low larval density were closer to those reared at intermediate larval density (1.53) than to those from the high larval density condition (2.71). However, for uninfected mosquitoes, this pattern of distribution was reversed, with the lower larval density being more distant from the intermediate condition (2.80), which then was closely related to the high density (1.96). Overall, there was a greater difference between the highest and lowest densities for uninfected individuals (3.34) than for infected mosquitoes (2.71).

**Fig. 5:**
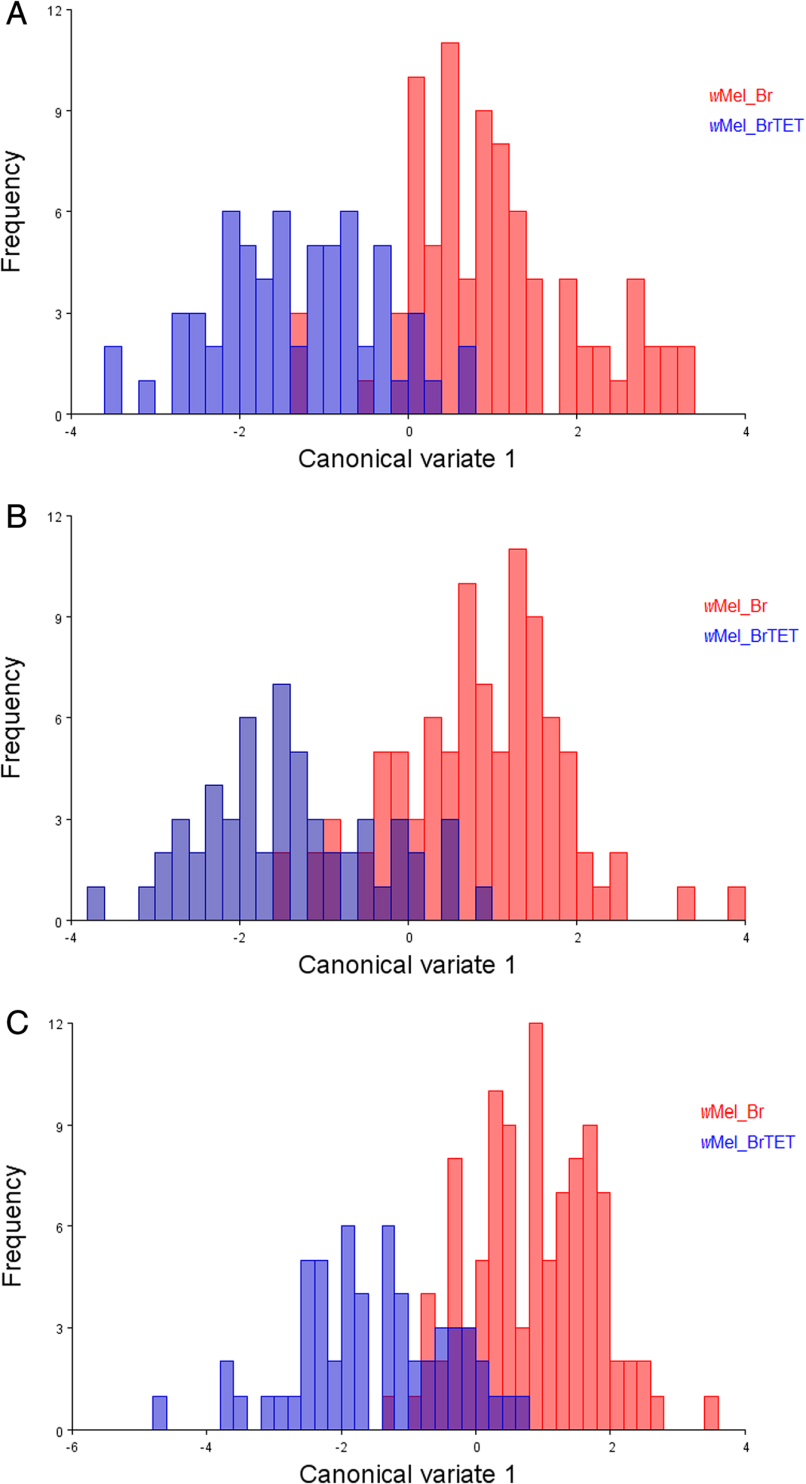
The influence of *w*Mel infection on wing shape in adult Brazilian *Ae. aegypti* females. Histograms displaying the main canonical variates for (**a**) low, (**b**) intermediate and (**c**) high densities without allometry, for *Wolbachia*-infected (*red*) and uninfected (*blue*) mosquitoes. Data were pooled from two independent biological replicates

Mahalanobis distances (MD) with 10,000 permutations were also computed for *Wolbachia*-infected and uninfected females within the same larval density and displayed as histogram of discriminant values (Fig. 5). For the lower larval density (Fig. 5a), allometry significantly accounted for 1.97 % of the observed effect (*P* = 0.0035), with a clear distinction between infected (*n* = 83) and uninfected (*n* = 64) individuals (MD = 2.34, *P* < 0.001). The same pattern of distinction was observed for the other larval densities. In the intermediate condition (Fig. 5b) infected individuals (*n* = 93) were separate from their uninfected counterparts (*n* = 56), (MD = 2.55, *P* < 0.001) after the removal of a significant allometric effect of 10.46 %; (*P* < 0.001). In the high-density condition (Fig. 5c), 102 infected and 57 uninfected individuals were analyzed and displayed a clear distinction (MD = 2.39, *P* < 0.001), which was present after the removal of a significant allometric effect (9.55 %, *P* < 0.001).

In an exploratory Principal Component Analysis (PCA), we found that a total of ten variables (dimensions) were required in order to explain 90.8 % of the variability associated with wing shape in our data. A large proportion of the total observed shape variation (62.3 %) was associated with the first three principal components (PC) based on the covariance matrix, which explained the majority of the observed differences between groups. PC1 (34.1 %) accounted for more variation than PC2 and PC3 combined (28.1 %). Deformation grids produced with the thin-plate spline showing wing shape temporal variations are depicted in Fig. 4c. For PC1, most of the variation can be explained by landmark 2 changing in the opposite direction to landmark 17 and 18. In PC2 the variation in wing shape can be attributed to landmark 2 moving into the opposite direction to the landmarks 12–16, whereas for PC3, it can be attributed to landmarks 12–16 moving towards the edge of the wing while landmarks 17–18 were positioned further towards the center.

### Glycogen content

**Fig. 6:**
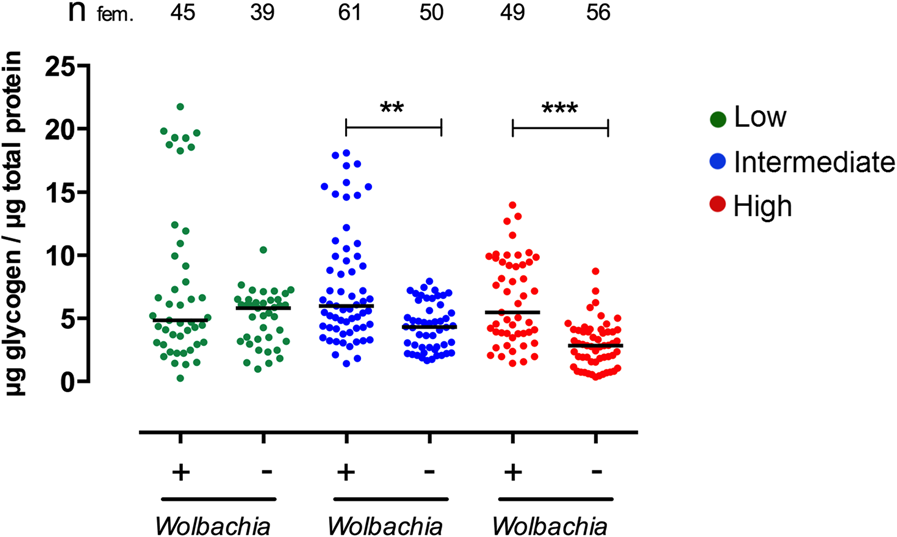
*w*Mel-infected Brazilian *Ae. aegypti* adult female mosquitoes have higher levels of glycogen after rearing at high larval density. Graphs depict the median glycogen levels of *w*Mel-infected (+) and uninfected (−) adult Brazilian *Ae. aegypti* female mosquitoes. Each circle represents a single adult female, while the horizontal black lines indicate the median glycogen content in each treatment. Green dots represent the lower density where there was no difference in the levels of glycogen between infected and uninfected females (Mann-Whitney U test, *U* = 795, *df* = 1, *P* = 0.4627). Blue and red dots depict the intermediate and higher densities, respectively, where *Wolbachia*-infected females had a higher level of glycogen (Intermediate: Mann-Whitney U test, *U* = 863, *df* = 1, *P* < 0.0001; High: Mann-Whitney U test, *U* = 536, *df* = 1, *P* < 0.0001). The total number of females (n_fem._) is indicated above each treatment. Data were pooled from two independent biological replicates

Comparison of glycogen levels across the six treatments using generalized linear models of regression revealed that both *Wolbachia* and larval density had a significant effect on mosquito glycogen reserves, but there was no effect of *Wolbachia* × larval density interaction (Table 4). Glycogen levels in uninfected mosquitoes decreased almost 50 % as larval density increased (Kruskal-Wallis *H* = 26.06, *df* = 2, *P* < 0.0001; range (median): 0.98–10.43 (5.82) μg (low density); 1.66–7.93 (4.30) μg (intermediate density); 0.34–8.70 (2.84) μg (high density). In contrast, median glycogen levels for *Wolbachia*-infected mosquitoes were more stable as larval density increased, with a non-significant 13 % increase (Kruskal-Wallis *H* = 1.485, *df* = 2, *P* = 0.4760); range (median): 0.24–21.74 (4.85) μg (low density); 1.41–18.12 (5.98 ) mg (intermediate density); 1.44–13.98 (5.48) μg (high density) (Fig. 6). There was no difference in glycogen levels between infected and uninfected individuals for the low-density condition (Mann-Whitney U test, *U* = 795, *df* = 1, *P =* 0.4627). However, glycogen levels were significantly higher in infected mosquitoes for the other two conditions (Intermediate: Mann-Whitney U test, *U* = 863, *df* = 1, *P <* 0.0001; High: Mann-Whitney U test, *U* = 536, *df* = 1, *P <* 0.0001).

**Table 4:** Analysis of the effects of *Wolbachia* infection and larval density on adult glycogen levels using generalized linear models

Source	*W*	*df*	*P*-value
Glycogen data			
Intercept	688.119	1	< 0.0001
*Wolbachia*	51.128	1	< 0.0001
Crowding	10.838	2	0.004
*Wolbachia* × Crowding	0.964	2	0.618

*W* Wald statistic, *df* degrees of freedom, *Wolbachia* stands for *Wolbachia* infection, crowding stands for larval density condition

## Discussion

We have examined how *w*Mel strain of *Wolbachia* affects *Ae. aegypti* larval development under different crowding conditions, and how this in turn alters adult mosquito morphology and energetic reserves (measured as glycogen content). Our results show that *w*Mel-infected larvae developed faster at higher densities for both males and females, with no effect under less crowded conditions in females. Infection also led to reduced body size at both high and low, but not intermediate density. Variation in wing shape was also observed between infected and uninfected mosquitoes due to larval density. *Wolbachia* infection also served to maintain stable glycogen levels at higher larval densities, whereas glycogen levels in uninfected mosquitoes were decreased.

### Larval development

Our observations of larval development dynamics corroborated previous reports [54, 55] showing a gradual increase in the time needed for both infected and uninfected larvae to reach pupal stage as the crowding conditions increased from low to high density.

In general, as larval density increased for both males and females, there were differences in the median development time due to infection at the intermediate and high densities, with infected individuals developing sooner than their uninfected counterparts. This result highlights the beneficial effect of *Wolbachia*, which acts as a mutualistic agent as conditions worsen and decreases development time for infected larvae. This could make the spread of the bacterium in the field much easier in places where field releases are taking place in Brazil [56], where a faster development rate makes juvenile individuals less susceptible to predators [57], a fact that can directly influence the population size in a given area through time.

These results differed from what was previously observed [58] on natural *Wolbachia* infections in *Ae. albopictus* where detrimental effects of infection on both males and females were observed as crowding increased. It is important to note that such differences may have occurred due to differences in strain biology or the fact that *w*Mel in *Ae. aegypti* is a transinfection [59]. In an Australian genetic background, larval density had no effect on development time in cohorts of *w*Mel-infected larvae, but there was a delayed development associated with infection when uninfected and infected larvae were reared together [25]. The conditions presented by these authors differ from what was tested here. Although they analyzed a broader spectrum of larval densities, ranging from 50 to 800 larvae per tray, this only represented a 16-fold increase in population density whereas here, we considered a 25-fold increase, which seems to be reasonable, considering the larval density of *Ae. aegypti* in the field [60, 61]. This highlights the importance of host genotype in host-symbiont interactions [62] and also the importance of taking into account different methodologies.

Previous studies have shown that *Wolbachia* infection can produce divergent phenotypic effects depending on the sex of the host, where *Wolbachia*-infected males of *Ae. albopictus* under high larval densities displayed a delayed development time compared to females [55, 58]. This is not unexpected given that *Wolbachia* biology is geared towards maternal transmission, with female hosts experiencing heavy selection pressure and male hosts left as little more than genetic dead-ends [63, 64]. As such, particular importance has to be given to sex-based analyses in *Wolbachia*-infected insects. Although females and males compete in the same environment, the latter given their reduced body mass, develop more quickly and utilize a fair amount of available resources, which indirectly influences the rate of population growth, potentially at the expense of the more epidemiologically important females [55].

### Morphometrics

When considering morphometric traits, we first analyzed wing size as a proxy of total body size [47]. There is evidence that greater female body size correlates to an increased ability to transmit pathogens [9], as well as increased fecundity, flight capacity, host-seeking in order to blood feed [65] and also resistance to desiccation in the field [66]. Previous work has shown that there is an inversely proportional correlation between wing size and larval density in *Ae. aegypti*, even when infected with *Wolbachia* [25] and this was similar to our results. We also observed that *w*Mel-infection was an important factor affecting wing size, with infected individuals displaying reduced body size for both low and high larval density conditions. This contrasts with what was observed in Australian *Ae. aegypti* mosquitoes infected with the same *Wolbachia* strain, where there was a beneficial effect of infection that resulted in larger wings [25]. Here, a large proportion of the differences between groups of infected and uninfected individuals could be attributed to a group of female mosquitoes displaying extreme values for body size (females that were either very small or very big), rather than the values around the actual median of the group.

Interestingly, we observed that wing size decreased to a similar degree for infected and uninfected mosquitoes as larval density increased (by 23.7 and 26 %, respectively): These data suggest that any density × *Wolbachia* effect affecting wing size and thus adult size, is likely to be only a minor effect. However, we did not compare this effect using a mixed cohort of *Wolbachia*-infected and uninfected individuals competing in the same tray. From the perspective of releasing *w*Mel-infected Brazilian females into the field for mosquito control, these results do not suggest that *w*Mel infection will serve as a significant disadvantage during competition against uninfected mosquitoes, as also shown elsewhere [25].

Variation in insect wing morphological traits such as the overall shape can affect dispersal [67] and the ability to find resources [68], as flight performance depends on thoracic mass associated with flight muscles, wing asymmetry and the ability to spatially and temporarily make use of variations in the prevailing winds, amongst other factors [69]. As such, wing morphology together with wing size are important factors to be taken into consideration when planning to rear competitive laboratory mosquitoes for future releases in the field [70]. Our results indicate that *Wolbachia* infection directly influences the wing shape of its host. Larval density also accounted for a degree of shape variation between treatments, with a pattern of proximity between groups as larval density increased (individuals from the low density were more closely related to those from the intermediate density, which were closer to females from the higher density treatment). Other studies support our observation that external factors can influence wing shape, as this variation was observed when comparing field mosquitoes against laboratory-reared mosquitoes. It was also observed when food availability was manipulated and also as an indicator of stress [70–72]. In our data, much of the variance in wing shape was associated with landmark 2 (costal notch) when analyzing the two main principal components that accounted for wing shape variation (PC1 and PC2). However, it is difficult to precisely say if this particular landmark can have direct implications in the fitness of field mosquitoes (given that this is a parameter influenced not only by just one variable) even though it was influenced by changes in food availability.

One important factor relating to wing size and its influence on host biology is the conditions in which the experiments were conducted (extrinsic factors), given the multifactorial nature of this trait. Some studies involved variation in temperature [9], relative humidity [66] and food availability [25, 71], while we considered different densities and food availability. Other authors analyzed the influence of body size on fecundity in *Ae. aegypti* [73] and other species [65]. Fundamentally, all these studies will provide a different outcome on how the conditions tested influenced wing size. However, all small mosquitoes are not identical. Lack of nutrients and high temperatures can both produce smaller mosquitoes, however, these mosquitoes are likely to have very different metabolic and nutritional profiles. As such, we reiterate the importance of a multifactorial approach, as presented here, to compare the overall fitness of an individual, instead of being based only in a single factor, i.e. size [74, 75].

### Glycogen

Insects in general are constantly expending energy; when they are not ingesting food, they are using their energy stocks in order to sustain many important bodily functions [76]. Triglycerides and glycogen are the two main forms of energy reserves that are stored in animal adipocyte cells. These are responsible for controlling the synthesis and utilization of energy reserves, but are also involved in the synthesis of most of the hemolymph proteins and circulating metabolites [11]. Previous work has shown that infection can increase levels of glycogen, as seen for *Plasmodium*-infected mosquitoes [77]. We saw that the *w*Mel-infected mosquitoes maintained stable levels of glycogen as larval density conditions became increasingly crowded, whereas uninfected mosquitoes experienced decreased levels of this energetic reserve as crowding stress increased. Higher glycogen levels under stressed conditions likely represents a fitness benefit due to *w*Mel infection, as seen elsewhere, with *Wolbachia* becoming a metabolic provisioning agent under conditions of high dietary iron stress in infected *Drosophila melanogaster* [78] whereas other *Wolbachia* strains display a range of nutritional mutualisms [79, 80]. Together, these traits could assist competitiveness in the field, especially given that it has already been demonstrated that in female *Ae. aegypti* mosquitoes, feeding on carbohydrates providing higher glycogen reserves, allows extended flight activities and is essential for survival in the absence of blood meals [10]. Interestingly, under high larval density, infected individuals presented reduced body size but higher glycogen levels. Previous studies with *Anopheles gambiae* have demonstrated that glycogen determines male mating success in a swarm, with the ability to initiate and sustain swarming being positively associated with carbohydrates reserves [10, 81] whereas for other species, there is a correlation between body size and mating competitiveness [15]; however this correlation is not always positive and there are cases where size is not a determinant of mating capacity [82].

### Implications for mosquito control

With field releases of the *w*Mel-infected *Ae. aegypti* mosquitoes just starting in Brazil, in areas that display great variability in their physical, human/mosquito population and demographic characteristics, it is critical to assess the effects of this bacterial strain in mosquitoes with a Brazilian genotype. Different *Wolbachia* strains can differently affect host physiology and this variability can be influenced by environmental conditions as well as host genetic background [27, 37, 83]. In the Brazilian genetic background, *w*Mel infection causes almost complete CI, has a high rate of maternal transmission and has no detrimental effect on fecundity, when females where reared under optimal conditions in a laboratory setting [37]. However, field conditions do not always provide the best-case scenario for insect development, as crowding and other factors can detrimentally affect fitness [19, 84–87]. As such, analysis of how *Wolbachia* affects mosquitoes reared under different density-dependent regimes will provide information that is vital to the planning stages of field releases of *Wolbachia*-infected mosquitoes in the field, along with an additional measure of infected mosquito competitiveness.

Our results indicate that the effects of *w*Mel on its host are variable depending on larval density conditions. Perhaps the most important benefit is the comparatively decreased development time, as insects that develop faster tend to have an advantage in the field in terms of exposure to larval predation [57], mating competition [88] and food availability [9]. Infected mosquitoes with faster development times also had elevated levels of glycogen, which would likely prove to be of great benefit in the field and could provide *Wolbachia*-infected mosquitoes with the fuel to fly further or longer and this could potentially aid in host or oviposition site location [10], as well as invasion. Both of these traits would likely improve adult competitiveness in the field.

In combination with the previously described physiological effects [37] it would be tempting to suggest that *w*Mel has an entirely beneficial effect in the Brazilian background. However, our data indicate that there are infection-dependent decreases in wing size and alterations in shape. These changes could affect flight and behaviours such as host-seeking which depends on flight [68]. The nature and extent of these effects are unclear and should be investigated further.

Nutritional benefits due to *Wolbachia* infection, as observed in our glycogen results are fairly common, but have not typically been associated with *Wolbachia* infection in *Ae. aegypti.* In terms of metabolic pathways, the *w*Mel strain is genetically limited, suggesting a reliance on host resources [89]. In *Ae. aegypti*, *w*Mel has been previously shown to deplete host cholesterol levels [76]. Our results indicate that *w*Mel has a complex metabolic relationship with its host, varying between parasitic and mutualistic, with this being the first example of a nutritional mutualism for this strain in mosquitoes [90, 91]. The provision of useful resources such as glycogen may also contribute to the faster development rates than we observed, especially given that similar effects on glycogen have been observed with the native *w*Flu strain in *Ae. fluviatilis* eggs [41].

Heavy fitness costs due to detrimental effects on the biology of *Wolbachia*-infected mosquitoes could hinder the spread of the bacterium in the field [40]. Our results indicate that such effects are not likely to occur for *w*Mel infections in Brazilian *Ae. aegypti*, particularly under conditions of high larval density and competition. Faster development rates and higher glycogen levels in infected in comparison to uninfected mosquitoes, may lead to increased competitiveness associated with infection and such beneficial fitness effects are desirable for the successful deployment of *Wolbachia* in the field [10, 92]. However these benefits could potentially be balanced by observed decreases in mosquito wing size, suggesting smaller body size and altered wing shape, which could affect flight, although not necessarily detrimentally.

The phenotypic effects we have observed are distinct from those previously observed in Australian mosquitoes, where this bacterium was first deployed as a biocontrol agent [25, 43]. This highlights the importance of investigating the effects of different host genetic backgrounds on *Wolbachia* infection, particularly in advance of releases of mosquitoes for vector control purposes. Our data provide additional information that can be used to inform release planning and design in *Wolbachia* population replacement mosquito control strategies in Brazil, where mosquitoes are being released into areas with large local mosquito populations [37] and frequent dengue transmission [93]. Specifically, they will be useful to developing an accurate picture of the fitness effects of *Wolbachia* infection, which is a requirement in the mathematical modeling used to predict whether *Wolbachia* can invade specific localities [56, 94]. These data could enable researchers to more accurately estimate the factors involved in *Wolbachia* release dynamics, such as the overall release period, number of mosquitoes releases and the distribution of releases within a field site, given our improved understanding of the competitiveness of *w*Mel-infected Brazilian *Ae. aegypti*.

## Conclusions

Our results suggest that the *w*Mel strain of *Wolbachia* can actually have beneficial effects on host physiology under certain conditions. We observed beneficial effects through decreased time to pupation and increased glycogen content under different larval crowding scenarios; however these were offset by a mild decrease in body size of female mosquitoes and distinct wing shapes associated with infection. These results, in combination with previous work, indicate that the *w*Mel strain of *Wolbachia* can likely be used successfully in open field releases in Brazil, as part of the Eliminate Dengue Program.

## Additional file

Additional file 1: Figure S1.
*w*Mel influences the median development time of juvenile males and females Brazilian *Ae. aegypti* mosquitoes. Box and whisker plots depicting the median pupation time in days for a, female; b, male *w*Mel-infected (+) and uninfected (−) *Ae. aegypti* mosquitoes. Green boxes represent the lower density, blue and red boxes the intermediate and higher densities, respectively. Female *w*Mel-infected mosquitoes developed faster than uninfected in the intermediate (Mann-Whitney U test, *U* = 16,957, *df* = 1, *P* = 0.0021) and high-density conditions (Mann-Whitney U test, *U* = 99,639, *df* = 1, *P* < 0.0001). Low density had no difference in the median development time between groups (Mann-Whitney U test, *U* = 9590, *df* = 1, *P* = 0.2404). For males, the same pattern occurred with the intermediate (Mann-Whitney U test, *U* = 26,399, *df* = 1, *P* = 0.0202) and high-density conditions (Mann-Whitney U test, *U* = 120,849, *df* = 1, *P* < 0.0001). While at the low-density condition, uninfected individuals developed faster (Mann-Whitney U test, *U* = 11,952, *df* = 1, *P* = 0.0016). Data were pooled from two independent biological replicates. The total number of mosquitoes analyzed (n_indiv._) is indicated above each group. (BMP 11747 kb)
