# Supplementary figures and images for: The influence of larval competition on Brazilian Wolbachia-infected Aedes aegypti mosquitoes
Source: Parasit Vectors. 2016 May 16;9:282. doi: 10.1186/s13071-016-1559-5 (PMC4869337; doi:10.1186/s13071-016-1559-5)

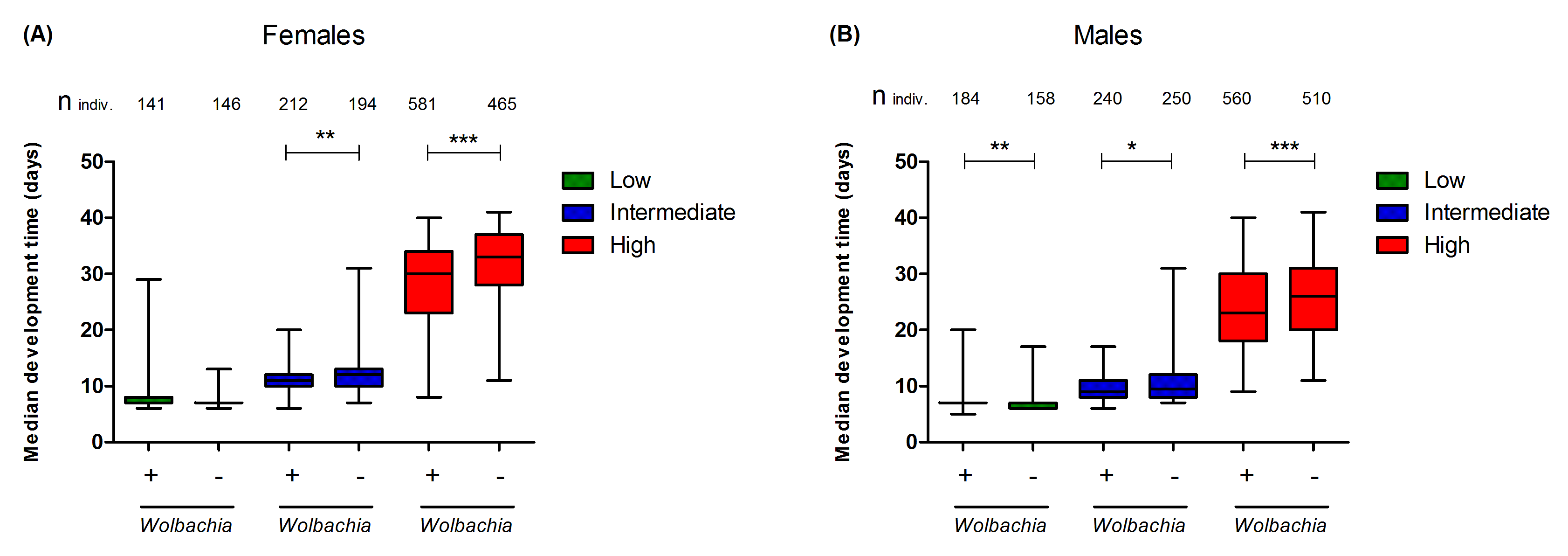

Supplement: Additional file 1: Figure S1. — wMel influences the median development time of juvenile males and females Brazilian Ae. aegypti mosquitoes. Box and whisker plots depicting the median pupation time in days for a, female; b, male wMel-infected (+) and uninfected (−) Ae. aegypti mosquitoes. Green boxes represent the lower density, blue and red boxes the intermediate and higher densities, respectively. Female wMel-infected mosquitoes developed faster than uninfected in the intermediate (Mann-Whitney U test, U = 16,957, df = 1, P = 0.0021) and high-density conditions (Mann-Whitney U test, U = 99,639, df = 1, P < 0.0001). Low density had no difference in the median development time between groups (Mann-Whitney U test, U = 9590, df = 1, P = 0.2404). For males, the same pattern occurred with the intermediate (Mann-Whitney U test, U = 26,399, df = 1, P = 0.0202) and high-density conditions (Mann-Whitney U test, U = 120,849, df = 1, P < 0.0001). While at the low-density condition, uninfected individuals developed faster (Mann-Whitney U test, U = 11,952, df = 1, P = 0.0016). Data were pooled from two independent biological replicates. The total number of mosquitoes analyzed (nindiv.) is indicated above each group. (BMP 11747 kb) [file 13071_2016_1559_MOESM1_ESM.bmp]
